# Suture trabeculotomy ab interno for secondary glaucoma in Japanese patients with Val30Met hereditary transthyretin amyloidosis

**DOI:** 10.1038/s41598-022-23150-8

**Published:** 2022-11-11

**Authors:** Takahiro Kawaji, Tomoki Sato

**Affiliations:** Sato Eye and Internal Medicine Clinic, 4160-270 Arao, Arao City, Kumamoto 860-0041 Japan

**Keywords:** Hereditary eye disease, Eye diseases, Glaucoma

## Abstract

We retrospectively evaluated surgical outcomes of suture trabeculotomy (SLOT) ab interno for secondary glaucoma in 18 eyes of 12 patients with hereditary transthyretin (ATTRv) amyloidosis with Val30Met mutation. SLOT ab interno was performed between May 2015 and January 2020. All the participants were followed up for at least 12 months. The primary outcome measure was Kaplan–Meier survival. Failure of this treatment was defined as an intraocular pressure (IOP) of ≥ 22 mmHg and a < 20% IOP reduction with or without medication or as additional operations needed to reduce IOP. The mean postoperative follow-up period was 3.5 years (1.2–6.1 years). The SLOT ab interno procedure alone was performed in 17 eyes (94%). Fifteen eyes (83%) had a 360° incision made in Schlemm’s canal and 3 eyes (17%) had a 180° incision performed. Cumulative survival values were 0.83, 0.63, and 0.22 at 1, 2, and 3 years, respectively. Ten eyes (56%) needed additional surgery, such as repeated SLOT ab interno, Ahmed glaucoma valve implantation, or MicroPulse transscleral cyclophotocoagulation. Our results here, as well as our previous results with trabeculectomy, suggest that SLOT ab interno may not have a sufficiently long-term effect on secondary glaucoma because of ATTRv amyloidosis.

Hereditary transthyretin (ATTRv) amyloidosis, also known as transthyretin-related familial amyloidotic polyneuropathy, is an autosomal dominant multisystem disease of adult onset characterized by systemic accumulation of mutant ATTR in organs and peripheral nerves. Of more than 130 ATTR mutations, Val30Met is highly prevalent in Portugal, Sweden, Japan, and other countries^1^. ATTRv amyloidosis, once believed to be restricted to an area of northern Portugal and a few other endemic areas, has now been documented as worldwide, with a global prevalence estimated at up to 38,000 persons^2,3^. Patients with ATTRv amyloidosis commonly have ocular manifestations, such as vitreous opacity, secondary open-angle glaucoma, abnormal conjunctival vessels, dry eye, loss of corneal sensitivity, neurotrophic corneal ulcers, anterior capsule opacity of the lens, retinal and/or choroidal vascular changes, pupillary light-near dissociation, irregular pupil, and optic neuropathy^4–6^. Among these manifestations, vitreous opacity and glaucoma are troublesome and can restrict daily lives of patients during the course of the illness^7–9^.

The pathophysiology of glaucoma in patients with ATTRv amyloidosis is still poorly understood. Previously reported data led to the accepted belief that ocular amyloid deposition may produce aqueous outflow route obstruction and/or perivascular amyloid deposition in conjunctival and episcleral tissues, which may be partly responsible for increased episcleral venous pressure and thus result in glaucoma^9–11^. Also, a few reports showed that glaucoma developed after 20-gauge^12^ or 25-gauge^13^ vitrectomy for vitreous opacity in ATTRv amyloidosis patients. These data suggested that a removal of vitreous body can cause diffusion of amyloid fibrils into the eye, especially the aqueous outflow route, which could be the cause of intraocular pressure (IOP) elevation after vitrectomy.

We previously reported the clinical features of our Japanese ATTRv amyloidosis patients who had secondary glaucoma, with glaucoma occurring in 12 (24%) of 49 patients: deposition of amyloid at the pupillary border, an irregular pupillary border, and vitreous opacity were strongly related to the development of glaucoma; and IOP was well controlled during the short follow-up period, a mean 1.2 years, after trabeculectomy with mitomycin C (MMC)^9^. However, our subsequent long-term follow-up results, a mean 5.4 years, indicated that the effect of trabeculectomy with MMC seemed to be limited to these glaucomatous eyes and that approximately half of these eyes needed additional surgery to reduce the IOP within two years after surgery. Bleb encapsulation, in particular, was seen in 47% of these eyes^14^. In addition to our study, certain basic research reports suggested that ATTRv aggregates in the conjunctiva can induce mild fibroblast activation, which may lead to poor results of trabeculectomy with MMC because of bleb-related complications^10,15,16^.

Trabecular-targeted minimally invasive glaucoma surgery (MIGS) has gained popularity for lowering IOP^17–20^. This surgery aims to relieve the resistance in the aqueous humor outflow by removing or incising the trabecular meshwork and inner layer of Schlemm's canal. The ab interno approach can preserve the conjunctiva, and surgical outcomes are not affected by conjunctiva-related complications such as trabeculectomized eyes with ATTRv amyloidosis. Our several previous reports concerned the clinical outcomes of suture trabeculotomy (SLOT) ab interno, one of the MIGS, that is used for open-angle glaucoma^21–23^. In the present study, we evaluated the outcomes of SLOT ab interno in secondary glaucoma associated with ATTRv amyloidosis.

## Results

### Clinical characteristics

Table 1 provides baseline demographic and clinical characteristics of patients in this study. This study included 18 eyes of 12 patients with secondary glaucoma associated with ATTRv amyloidosis with an Val30Met mutation who underwent a SLOT ab interno. The mean age was 48.3 ± 5.6 years at the time of SLOT ab interno. The mean time from the onset of systemic symptoms to any kind of glaucoma surgery, such as SLOT ab interno or trabeculectomy, was 14.5 years All patients underwent liver transplantation at the mean age of 33.5 ± 5.9 years, which was an average of 0.9 ± 1.5 years after the initial systemic symptoms. The mean follow-up period after SLOT ab interno was 3.5 years.

**Table 1:** Baseline demographic and clinical characteristics of patients with hereditary transthyretin amyloidosis with the Val30Met mutation.

Characteristic	Value
No. of eyes (patients)	18 (12)
Sex (male/female), n	5/7
Age at onset of systemic disease, y (range)	32.7 (27–42)
Age at liver transplantation, y (range)	33.5 (28–42)
Time from onset of systemic disease to glaucoma surgery, y (range)	14.5 (4–24)
Age at surgery, y (range)	48.3 (31–54)
Follow-up period after surgery, y (range)	3.5 (1.2–6.1)
Prior surgery, no. (%) of eyes	
Vitrectomy	7 (39%)
Cataract surgery	5 (28%)
Glaucoma surgery	
Trabeculectomy	3 (17%)
Cyclophotocoagulation by Cyclo G6 MicroPulse P3	3 (17%)
Intraocular pressure, mmHg (range)	29.9 (25–41)
Number of glaucoma medications, n (range)	4.9 (4–6)
Best corrected visual acuity, logMAR (range)	0.05 (-0.7–1.8)

*logMAR* logarithm of the minimum angle of resolution.

### Previous ocular surgery

Seven eyes (39%) underwent vitrectomy an average of 1.2 ± 1.9 years (range, 0.2–5.1) before SLOT ab interno. Five eyes (28%) had glaucoma surgery before SLOT ab interno: two eyes had trabeculectomy with MMC, two eyes had MP-TSCPC, and one eye had trabeculectomy with MMC and MP-TSCPC. Cataract surgery by phacoemulsification of the crystalline lens was performed in five eyes (28%); it was combined with vitrectomy in four eyes, and one eye had cataract surgery alone.

### Outcomes

The SLOT ab interno procedure alone was performed in 17 eyes (94%), and SLOT ab interno combined with phacoemulsification was performed in one eye (6%). Fifteen eyes (83%) had a 360° incision in Schlemm’s canal and three eyes (17%) had a 180° incision, including an upper-180° incision in one eye and a lower-180° incision in two eyes.

**Figure 1:**
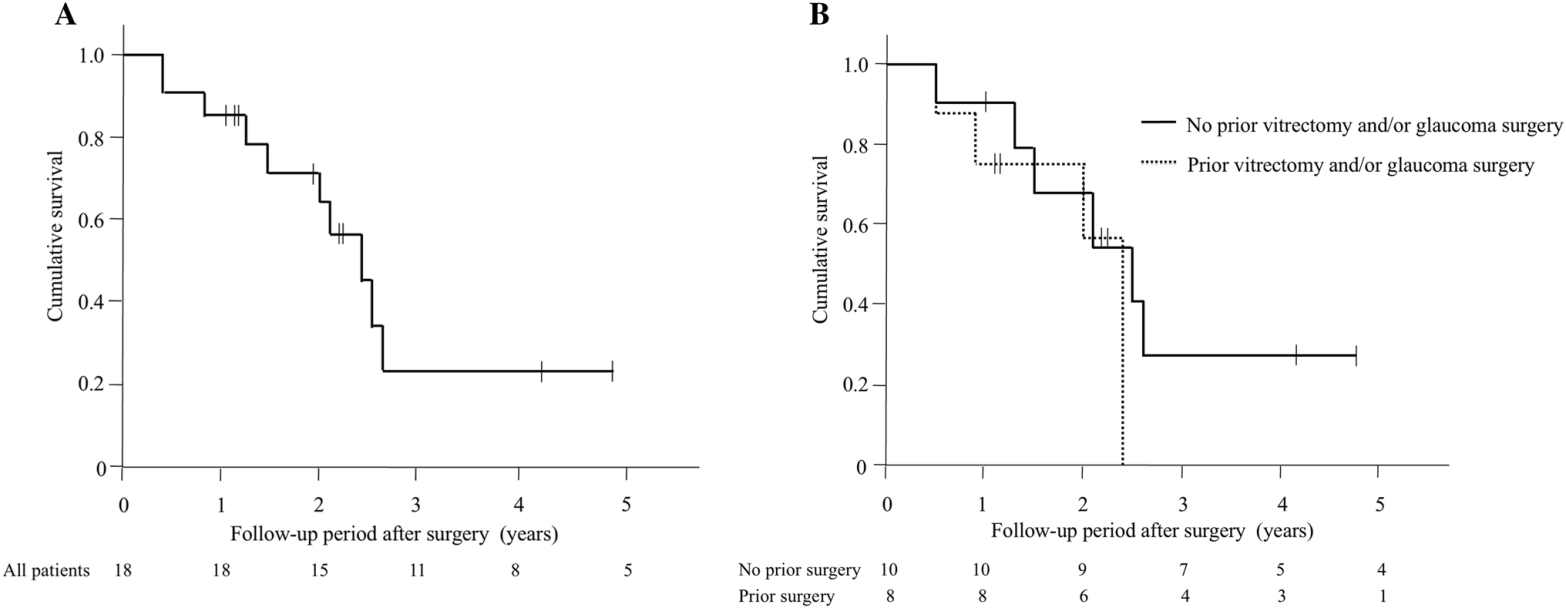
Survival analysis and comparison of glaucoma treatments. (**A**) Kaplan–Meier survival analysis of 18 eyes treated with suture trabeculotomy ab interno in all eyes. (**B**) Comparison of eyes with or without previous glaucoma surgery and/or vitrectomy. Treatment failure was defined as an intraocular pressure (IOP) value of ≥ 22 mmHg and a < 20% IOP reduction with or without medication on two consecutive visits, or as additional operations needed to reduce IOP. The small vertical dashes along the curves represent times of censored observations.

Figure 1 presents the result of Kaplan–Meier survival analysis of 18 eyes that underwent SLOT ab interno. According to treatment success criteria, cumulative survival values were 0.83 at 1 year, 0.63 at 2 years, and 0.22 at 3 and 4 years (Fig. 1A). Eight eyes were censored because regular follow-up ended. Ten eyes (56%) of eight patients were classified as surgical failures. Success rates were similar among eyes with or without previous glaucoma surgery (*P* = 0.45), previous vitrectomy (*P* = 0.38), and previous glaucoma surgery and/or vitrectomy (*P* = 0.46) (Fig. 1B). The results of the Cox proportional hazard model analysis indicated that was age, sex, age at onset of systemic disease, time from onset of systemic disease to glaucoma surgery, prior trabeculectomy, prior vitrectomy, and preoperative IOP were not significantly associated with success (*P* > 0.05). When using an IOP criterion of 6–18 mm Hg, cumulative survival values were 0.78 at 1 year, 0.43 at 2 years, and 0.22 at 3 and 4 years.

The mean IOP and the mean number of antiglaucoma medications were reduced from the preoperative values 29.9 ± 4.5 mmHg and 4.9 ± 0.8 to 15.1 ± 4.1 mmHg and 2.9 ± 1.6 at the final visit, respectively. The mean postoperative IOP and the number of medications were significantly reduced compared with the baseline values at all time points (Table 2).

**Table 2:** Intraocular pressure and number of antiglaucoma medications used in patients.

	IOP, mmHg (range)	*P* values^#^	Number (range) of antiglaucoma medications	*P* values^#^	*n*
Before surgery	29.9 (25–41)		4.9 (4–6)		18
1 month	12.1 (5–17)	< 0.0001	0.6 (0–3)	< 0.0001	18
3 months	12.4 (5–19)	< 0.0001	0.9 (0–3)	< 0.0001	18
6 months	13.4 (7–33)	< 0.0001	1.3 (0–3)	< 0.0001	18
12 months	14.1 (7–20)	< 0.0001	1.7 (0–4)	< 0.0001	18
24 months	19.4 (11–30)	< 0.0001	2.8 (0–4)	0.0004	14
	*P* < 0.0001*		*P* < 0.0001*		

*IOP* intraocular pressure.

#Calculated using the t test between the preoperative and respective time period values.

*Calculated using the mixed-effects regression model.

The mean postoperative BCVA did not change significantly (*P* = 0.87). The BCVA decreased by two or more lines from baseline in three eyes of two patients. The causes of these decreases were the progression of visual field defects and macula edema in two eyes of one patient and the progression of vitreous opacity in one eye of another patient.

### Interventions and complications

Table 3 summarizes the intra- and postoperative complications. The hyphema spontaneously resolved by two weeks in all eyes. Macula edema occurred in two eyes of one patient and resolved after a sub-Tenon injection of triamcinolone acetonide in one eye and non-steroidal anti-inflammatory eye drops used in one eye.

**Table 3:** Intraoperative and Postoperative Complications.

Outcome	
Intraoperative blood reflux, *n* (%)	18 eyes (100%)
Hyphema with niveau formation, *n* (%)	7 eyes (38%)
Transient IOP elevation of > 30 mmHg, *n* (%)	5 eyes (28%)
Macular edema	2 eyes (11%)
Additional surgical procedures, *n* (%)	10 eyes (56%)
SLOT ab interno, *n* (%)	7 eyes (39%)
Ahmed glaucoma valve implantation, *n* (%)	7 eyes (39%)
Cyclophotocoagulation via Cyclo G6 MicroPulse P3, *n* (%)	5 eyes (28%)
Cataract surgery, *n* (%)	4 eyes (22%)
Vitrectomy for vitreous opacity, *n* (%)	1 eye (6%)
Vitrectomy for retinal detachment, *n* (%)	1 eye (6%)
Sub-Tenon triamcinolone acetonide	1 eye (6%)

*IOP* intraocular pressure, *SLOT* suture trabeculotomy.

Ten eyes (56%) of eight patients required subsequent surgery to reduce IOP as follows: one eye, SLOT ab interno; one eye, SLOT ab interno twice; two eyes, Ahmed glaucoma valve implantation (AGV); one eye, SLOT ab interno and AGV twice; two eyes, SLOT ab interno, AGV, and MP-TSCPC; one eye, SLOT ab interno, AGV, and MP-TSCPC twice; one eye, SLOT ab interno three times and MP-TSCPC five times; and one eye, AGV and MP-TSCPC. The mean time from initial SLOT ab interno to additional glaucoma surgery was 1.7 years (range, 0.5–2.7 years). The tube in the AGV implantation was placed in the posterior chamber in all cases.

Two eyes (11%) underwent vitrectomy after SLOT ab interno because of the progression of vitreous opacity in one eye and rhegmatogenous retinal detachment in one eye. Cataract surgery by phacoemulsification of the crystalline lens was performed in one eye (6%).

## Discussion

Before the utilization of liver transplantation, early-onset ATTRv amyloidosis (before the age of 50 years) was fatal, with an expected survival of about ten years from disease onset^1^. Liver transplantation was introduced in the 1990s as the first treatment for ATTRv amyloidosis, and it persisted for two decades as the only therapy available with clear clinical evidence of benefit. In the last decade, various disease-modifying therapies have been developed. In Japan, the TTR stabilizer (tafamidis [Vyndaqel]; Pfizer, New York, NY, USA) and TTR mRNA silencers (patisiran [Onpattro]; Alnylam, Cambridge, MA, USA) are approved for clinical use^24^. These drugs, in addition to liver transplantation, can reduce the systemic production of ATTRv and significantly improve the survival and quality of life of these patients. However, ocular tissues—the retinal and ciliary pigment epithelia—also synthesize ATTRv^25,26^, and these drugs do not affect ocular ATTRv production and ocular complications. This continuous production of ATTRv may explain the difficulty in reducing the IOP and why the course of glaucoma in these patients is accelerated^8^.

Our present study providing mid-term follow-up results indicated that the effect of SLOT ab interno, as well as that of trabeculectomy with MMC, seemed to be restricted to secondary glaucoma associated with ATTRv amyloidosis. The cumulative probability of treatment success in our study was 0.83 at 1 year, 0.63 at 2 years, and 0.22 at 3 and 4 years. We classified ten eyes (56%) of eight patients as surgical failures. In our previous results of trabeculectomy with MMC for patients with ATTRv Val30Met, the cumulative survival values were 0.88 at 1 year, 0.81 at 2 years, 0.63 at 3 years, and 0.17 at 4 years. We classified 8 (50%) of 16 eyes as surgical failures^14^. In both studies, approximately half of the eyes needed additional surgical procedures within two years after surgery.

Although a trabecular-targeted MIGS, such as SLOT ab interno in this study, can relieve outflow resistance at the trabecular meshwork, 25–50% of the total outflow resistance is still located at the conventional outflow pathway distal to Schlemm’s canal^27^. Thus, effective functioning of the distal outflow tract is quite important to lower the IOP after MIGS. Given the pathophysiology of secondary glaucoma associated with ATTRv amyloidosis, amyloid deposition in the trabecular meshwork would play a predominant role. Therefore, a trabecular-targeted MIGS that removes the affected trabecular meshwork seemed a logical strategy. However, our insufficient results suggested that continuous amyloid deposition in the distal outflow tract and/or perivascular amyloid deposition in episcleral tissues may result in IOP increases even after SLOT ab interno. Barbosa et al.^28^ described a successful case report using the Kahook Dual Blade excisional goniotomy in a patient with ATTRv amyloidosis, but the follow-up period was only 6 months.

In the present study, ten eyes (56%) of eight patients required additional surgery to reduce the IOP. We performed SLOT ab interno as a first additional surgery, because SLOT ab interno is less invasive than other surgeries and is short operation time, and we expected that this procedure could allow removal of slight amyloid deposition around the trabeculotomy cleft or collector channnels, if it existed, which may reduce the IOP. Although the AGV was needed within one year after additional SLOT ab interno in five of seven eyes, IOPs remained stable in the other two eyes. In the two eyes, additional SLOT might wash out the amyloid deposition in the distal outflow tract, especially around the trabeculotomy cleft or collector channels. No serious complication related to additional SLOT ab interno procedures was seen.

In the recent decade, two different types of long-tube glaucoma drainage devices for refractory glaucoma were approved for use in Japan: the Baerveldt glaucoma implant (BGI; Abbott Medical Optics, Abbott Park, IL, USA) in 2012 and the AGV in 2014. Recently, Kakihara et al.^29^ suggested that BGI surgery may now be the optimal treatment for secondary glaucoma in Japanese patients with ATTRv amyloidosis, in that it produced good outcomes in five eyes of four patients with a mean of 4.4 years of follow-up. Marta et al.^30^ also showed that implantation of the AGV is a safe and effective option in secondary glaucoma in Portuguese patients, with excellent success and low complication rates in 114 eyes of 87 patients with a mean 3.8 years of follow-up. In that report, Marta et al. stated that although they published no data on the effects of trabeculectomy on these patients with glaucoma, their experience until 2009 indicated to them that trabeculectomy was not a good option, because the success rate was quite low. They reported better cumulative probability of AGV treatment success with rates of 0.98 at 1 year, 0.97 at 2 years, 0.95 years at 3 years, and 0.89 at 4 years. They also suggested that the AGV may be superior to filtering surgery, possibly because these patients had a modified and fragile conjunctiva as well as associated amyloid deposition, previous surgeries, and the cumulative use of multiple eye drops (not only for glaucoma but also for dry eye). In our study, we implanted the AGV after SLOT ab interno in seven eyes, and IOPs were controlled at < 21 mmHg at the final visit in all eyes except one (detailed data not shown). We also performed MP-TSCPC on eyes that received prior incisional glaucoma surgery, such as SLOT ab interno and AGV, after consulting with the patients. Given the above results, long-tube glaucoma surgeries seem to be preferable for secondary glaucoma associated with ATTRv patients. However, SLOT ab interno and any type of trabecular-targeted MIGS, which are safer and less invasive procedures, may be used before long-tube glaucoma surgeries for young or hesitant patients for long-tube surgeries especially with careful explanation about poor long-term effect.

In the present study, we utilized a 360° incision in Schlemm’s canal in 15 eyes (83%) and a 180° incision in three eyes (17%). We chose the SLOT ab interno for our patients, because we believed it reasonable to hypothesize that the IOP reduction would be proportional to the extent of the diseased trabecular meshwork and SLOT ab interno procedure is eager to perform a longer incision than that used in several types of trabecular-targeted MIGS, including trabecular ablation via the Trabectome, iStent, Kahook Dual Blade, and microhook ab interno trabeculotomy. Although the most appropriate length of Schlemm’s canal incisions is still controversial, we recently reported that the different lengths and locations of Schlemm’s canal incisions during SLOT ab interno for open angle glaucoma, including a 360° incision, an upper-180° incision, and a lower-180° incision, did not affect both the IOP reduction and the need for medications during a 12-month follow-up.^23^ Therefore, we currently use the 180° incision in Schlemm’s canal for the initial surgery; in this study, we used this incision in three recent cases.

Our study has several limitations that should be take into account when interpreting its results. The major limitations are its retrospective design, the lack of a control group and inclusion of eyes with prior ocular surgeries. Confounding factors and bias are inherent to retrospective studies. To minimize these limitation, we will need to conduct a multicenter study and selected strict inclusion and exclusion criteria. The inclusion of both eyes of a patient and various follow-up periods may also have introduced bias, although we minimized this by using a mixed-effects regression model. Although this disease is rare and the number of patients is small in this single-center study, further studies with larger cohorts and longer follow-up using SLOT ab interno or other trabecular-targeted MIGS devices are warranted.

In summary, as a consequence of the significantly improved survival of patients with ATTRv amyloidosis, glaucoma is becoming a more common serious complication. Our results suggest that SLOT ab interno, as well as trabeculectomy with MMC, may not have a sufficiently long-term effect on secondary glaucoma resulting from ATTRv amyloidosis.

## Methods

### Study design and patients

We retrospectively reviewed the medical records of patients with secondary glaucoma associated with ATTRv amyloidosis who had the Val30Met mutation and who underwent a SLOT ab interno at the Sato Eye and Internal Medicine Clinic (Arao City, Kumamoto, Japan) between May 2015 and January 2020. Eyes that had a postoperative follow-up time of less than 1 year were excluded. All records were evaluated for clinical signs of ATTRv amyloidosis, such as polyneuropathy, autonomic dysfunction, and visual disturbance, after which patients had a definitive diagnosis on the basis of amyloid deposits in biopsy samples and genetic investigations. The age at onset of ATTRv amyloidosis was defined as the time when the patient first complained of the typical symptoms just mentioned. The study protocol adhered to the principles of the Declaration of Helsinki and was approved by the Institutional Review Board and the Ethics Committee of the Sato Eye and Internal Medicine Clinic (IRB No. 202103). Before surgery, all patients gave written informed consent for the operation and this study.

### Outcome measures

The main outcome measure was success or failure of SLOT ab interno as determined according to the Kaplan–Meier analysis. Surgical success was defined as an IOP value between 6 and 21 mmHg with a postoperative IOP reduction of at least 20% with or without antiglaucoma medication. A surgical failure was defined as not meeting a success criterion at two consecutive follow-up visits or as a need for additional glaucoma operations to reduce the IOP. Additional analysis using alternate IOP targets of 6–18 mm Hg was performed. Secondary outcome measures included the mean IOP change at each visit, complications, and postoperative interventions required. IOP data obtained less than one month after SLOT ab interno were excluded because of IOP fluctuations. One surgeon (T.S.) performed all operations; he used the surgical procedure described here.

### Surgical technique

The SLOT ab interno technique used here was previously described^23^. In brief, after a 1.7-mm temporal corneal incision, Schlemm’s canal on the nasal side was incised at 15° by means of a microhook needle (HS-2167; Handaya, Tokyo, Japan). We used a 360° incision made in Schlemm’s canal in most cases. For an upper- or lower-180° SLOT ab interno procedure, the suture was inserted into nearly half of Schlemm’s canal and was pulled out after an inspection was performed with a surgical gonio lens (AU-700-476; Ocular Instruments, Bellevue, WA, USA) to ensure that the tip of the suture was located at the opposite side. If needed, a standard phacoemulsification with intraocular lens implantation was achieved through the same incision or a new upper corneal incision, with the phacoemulsification and lens implantation being performed after the SLOT ab interno. For eyes that required operations to reduce the IOP after the SLOT ab interno, we used alternatives such as repeated SLOT ab interno, Ahmed glaucoma valve implantation (AGV; New World Medical, Rancho Cucamonga, CA, USA), or MicroPulse transscleral cyclophotocoagulation (MP-TSCPC; Cyclo G6 MicroPulse P3; IRIDEX Corporation, Mountain View, CA, USA).

### Data collection and statistical analysis

All patients were examined on postoperative days one, two, and three, and then every one–two weeks for one month, monthly until month six, and every two–three months after that. At every visit, the IOP was measured via the Goldmann tonometer. The best corrected decimal visual acuity was measured by means of standard Landolt C charts; values were converted to logMAR (logarithm of the minimum angle of resolution).

The JMP statistical package (version 15; SAS Institute Inc., Cary, NC, USA) was used to analyze all data. We utilized the Kaplan–Meier survival analysis to determine the cumulative probability of success, and a log-rank test for comparisons among groups. The preoperative IOP and IOPs measured at 1 month, 3 months, 6 months, 12 months, and 24 months postoperatively were compared using a mixed-effects regression model in which each patient’s identification number was regarded as a random effect and the time period was regarded as a fixed effect; this was followed by a *t* test for the post hoc comparisons between groups. Postoperative changes in the number of antiglaucoma medications were also assessed using the mixed-effects regression model. Continuous data were expressed as means ± standard deviation.A Cox proportional hazard regression analysis was used to determine predictive factors for failure. The statistical significance value was set at *P* < 0.05.

## Data Availability

The datasets generated during and/or analysed during the current study are available from the corresponding author on reasonable request.

## References

[1] Adams D (2021). Expert consensus recommendations to improve diagnosis of ATTR amyloidosis with polyneuropathy. J. Neurol..

[2] Schmidt HH (2018). Estimating the global prevalence of transthyretin familial amyloid polyneuropathy. Muscle Nerve.

[3] Waddington-Cruz M (2019). Epidemiological and clinical characteristics of symptomatic hereditary transthyretin amyloid polyneuropathy: A global case series. Orphanet J. Rare Dis..

[4] Sandgren O (1995). Ocular amyloidosis, with special reference to the hereditary forms with vitreous involvement. Surv. Ophthalmol..

[5] Ando E (1997). Ocular manifestations of familial amyloidotic polyneuropathy type I: Long-term follow up. Br. J. Ophthalmol..

[6] Reynolds MM (2017). Ocular manifestations of familial transthyretin amyloidosis. Am. J. Ophthalmol..

[7] Koga T (2003). Vitreous opacities and outcome of vitreous surgery in patients with familial amyloidotic polyneuropathy. Am. J. Ophthalmol..

[8] Hara R (2010). Impact of liver transplantation on transthyretin-related ocular amyloidosis in Japanese patients. Arch. Ophthalmol..

[9] Kimura A (2003). Secondary glaucoma in patients with familial amyloidotic polyneuropathy. Arch. Ophthalmol..

[10] Futa R (1984). Familial amyloidotic polyneuropathy: Ocular manifestations with clinicopathological observation. Jpn. J. Ophthalmol..

[11] Silva-Araujo AC (1993). Aqueous outflow system in familial amyloidotic polyneuropathy, Portuguese type. Graefes Arch. Clin. Exp. Ophthalmol..

[12] Beirão NM (2012). Vitreous surgery impact in glaucoma development in liver transplanted familial amyloidosis ATTR V30M Portuguese patients. Amyloid.

[13] Kakihara S (2020). Small gauge vitrectomy for vitreous amyloidosis and subsequent management of secondary glaucoma in patients with hereditary transthyretin amyloidosis. Sci. Rep..

[14] Kawaji T (2014). Long-term outcomes and complications of trabeculectomy for secondary glaucoma in patients with familial amyloidotic polyneuropathy. PLoS ONE.

[15] Ophir A (1992). Encapsulated filtering bleb. A selective review—new deductions. Eye.

[16] Monteiro FA (2006). Activation of ERK1/2 MAP kinases in familial amyloidotic polyneuropathy. J. Neurochem..

[17] Malvankar-Mehta MS (2015). iStent with phacoemulsification versus phacoemulsification alone for patients with glaucoma and cataract: A meta-analysis. PLoS ONE.

[18] Leonard KS (2013). Preclinical investigation of ab interno trabeculectomy using a novel dual-blade device. Am. J. Ophthalmol..

[19] Tanito M (2017). Short-term results of microhook ab interno trabeculotomy, a novel minimally invasive glaucoma surgery in Japanese eyes: Initial case series. Acta Ophthalmol..

[20] Grover DS (2014). Gonioscopy-assisted transluminal trabeculotomy, ab interno trabeculotomy: Technique report and preliminary results. Ophthalmology.

[21] Sato T, Kawaji T, Hirata A, Mizoguchi T (2018). 360-degree suture trabeculotomy ab interno to treat open-angle glaucoma: 2-year outcomes. Clin. Ophthalmol..

[22] Sato T, Kawaji T, Hirata A, Mizoguchi T (2018). 360-degree suture trabeculotomy ab interno with phacoemulsification in open-angle glaucoma and coexisting cataract: A pilot study. BMJ Open Ophthalmol..

[23] Sato T, Kawaji T (2021). 12-month randomised trial of 360° and 180° Schlemm's canal incisions in suture trabeculotomy ab interno for open-angle glaucoma. Br. J. Ophthalmol..

[24] Adams D, Koike H, Slama M, Coelho T (2019). Hereditary transthyretin amyloidosis: A model of medical progress for a fatal disease. Nat. Rev. Neurol..

[25] Cavallaro T (1990). The retinal pigment epithelium is the unique site of transthyretin synthesis in the rat eye. Invest. Ophthalmol. Vis. Sci..

[26] Kawaji T (2005). Transthyretin synthesis in rabbit ciliary pigment epithelium. Exp. Eye Res..

[27] Rosenquist R (1989). Outflow resistance of enucleated human eyes at two different perfusion pressures and different extents of trabeculotomy. Curr. Eye Res..

[28] Barbosa NB, Grippo TM (2020). Excisional goniotomy with Kahook Dual Blade in a patient with glaucoma secondary to Transthyretin Amyloidosis. Am. J. Ophthalmol. Case Rep..

[29] Kakihara S (2020). Baerveldt glaucoma drainage implant surgery for secondary glaucoma in patients with transthyretin-related familial amyloid polyneuropathy. Jpn. J. Ophthalmol..

[30] Marta A, Vieira R, Figueiredo A (2022). Ahmed valve for secondary glaucoma in patients with hereditary transthyretin amyloidosis. Eye.

